# Utility of WHOQOL-BREF in measuring quality of life in Sickle Cell Disease

**DOI:** 10.1186/1477-7525-7-75

**Published:** 2009-08-10

**Authors:** Monika R Asnani, Garth E Lipps, Marvin E Reid

**Affiliations:** 1 Sickle Cell Unit, Tropical Medicine Research Institute, University of the West Indies, Mona Campus, Kingston 7, Jamaica; 2 Department of Psychology, Sociology and Social Work, University of the West Indies, Mona Campus, Kingston 7, Jamaica

## Abstract

**Background:**

Sickle cell disease is the commonest genetic disorder in Jamaica and most likely exerts numerous effects on quality of life (QOL) of those afflicted with it. The WHOQOL-Bref, which is a commonly utilized generic measure of quality of life, has never previously been utilized in this population. We have sought to study its utility in this disease population.

**Methods:**

491 patients with sickle cell disease were administered the questionnaire including demographics, WHOQOL-Bref, Short Form-36 (SF-36), Flanagan's quality of life scale (QOLS) and measures of disease severity at their routine health maintenance visits to the sickle cell unit. Internal consistency reliabilities, construct validity and "known groups" validity of the WHOQOL-Bref, and its domains, were examined; and then compared to those of the other instruments.

**Results:**

All three instruments had good internal consistency, ranging from 0.70 to 0.93 for the WHOQOL-Bref (except the 'social relationships' domain), 0.86–0.93 for the SF-36 and 0.88 for the QOLS. None of the instruments showed any marked floor or ceiling effects except the SF-36 'physical health' and 'role limitations' domains. The WHOQOL-Bref scale also had moderate concurrent validity and showed strong "known groups" validity.

**Conclusion:**

This study has shown good psychometric properties of the WHOQOL-Bref instrument in determining QOL of those with sickle cell disease. Its utility in this regard is comparable to that of the SF-36 and QOLS.

## Background

Sickle cell disease (SCD) is the commonest genetic disorder in Jamaica with the sickle hemoglobin (HbS) gene being present in about 10% of the population. It includes a variety of pathological conditions [1] and affects the individual throughout their life cycle. In Jamaica, SCD has become a significant indirect cause of maternal mortality [2] and contributes as a causative factor to 0.7% of cases of chronic renal failure [3]. It has also been presented as one of the 10 most common causes of sudden death in Jamaica accounting for 2.5% of cases [4]. Among those with homozygous sickle cell disease (SS) in Jamaica, there is a 50% survival to 30 to 40 years. Median survival is calculated at 53 years for men and 58.5 for women [5].

SCD carries a huge psychosocial burden impacting on physical, psychological, social and occupational well-being as well as levels of independence [6-14]. Psychological complications in patients with SCD mainly result from the impact of pain and symptoms on their daily lives and society's attitudes towards them [15-17]. Generally, there is increased psychological morbidity such as depression and poor coping [9,10,18-22], and poorer quality of life (QOL) [9,14,23].

The Short-Form 36 (SF-36) has been validated for measuring QOL in this population [24], but the World Health Organization Quality of Life- BREF (WHOQOL-BREF) has never been studied in these patients. Whereas the SF-36 provides some measure of functional status along with health related QOL, the WHOQOL-BREF measures relatively broader and totally subjective domains [25-27]. Its particular strength lies in the fact of its cross-cultural development employing elements of emic and etic perspectives [28], and as the Jamaican population represents a forging of different ethnicities as well as distinct cultures [29], the WHOQOL-Bref may prove to be a stronger measure of QOL. The Flanagan's quality of life scale (QOLS) is a generic scale but has had particular adaptation for use among persons with chronic diseases [30]. A comparison of these generic instruments will allow further study of their possible weaknesses and strengths. Therefore, the specific aims of this study are to: i) assess the properties of WHOQOL-BREF in SCD; and ii) compare the properties of the WHOQOL-BREF, SF-36 and QOLS in SCD.

In the current study we expected that the WHOQOL -physical subscale should be strongly correlated (r ≥ 0.50) with SF-physical health, role limitations and total scores, but less correlated (r ≤ 0.30) with SF-mental health scores as this subscale assesses the physical state of patient's quality of life. We expect a smaller correlation (r ≥ 0.30) with clinical indicators such as haemoglobin and serum lactate dehydrogenase (LDH). WHOQOL-psychological health domain may be strongly correlated (r ≥ 0.50) with the SF-mental health, SF-36 total score and the QOLS, but only moderately (r ≤ 0.30) with SF-physical health and role limitations subscales. The WHOQOL-social relations and environment subscales are expected to be strongly correlated (r ≥ 0.50) with the SF-mental health subscale, the SF-36 total score and the QOLS scale, but less (r ≤ 0.30) with the SF-physical and role limitations subscales, and (r ≤ 0.30) with haemoglobin and LDH. Finally, we expect the total WHOQOL-Bref score to be strongly correlated (r ≥ 0.50) with the total SF-36 and QOLS scores.

## Methods

### Study population

This was designed as a cross-sectional study. The Sickle Cell Unit (SCU) in Kingston operates Jamaica's only comprehensive sickle cell centre. All adults over the age of 18 years, registered at the SCU for at least 1 year, and presenting for health maintenance visit from January to June 2005 were invited to take part and none declined.

### Study Instruments

The SF-36, QOLS and WHOQOL-BREF (U.K.version) were interviewer-administered (as only about 80% of Jamaicans are considered to be functionally literate [31]) to all participants after they had signed an informed consent form. Data were also collected on age, sex, genotype, marital status, level of education achieved, employment status and occupation.

### Study Instruments

In past research, the WHOQOL-BREF has shown good to excellent reliability and validity, and has four domains: physical, psychological, social and environment [32]. Thomas et al [14], in their qualitative work with patients who have SCD, have identified themes that are quite similar to the core domains of the WHOQOL.

The psychometric properties of the SF-36 have been studied in the Jamaican population with SCD and it shows a slightly different component structure [33] yielding three distinct subscales: physical health, mental health and role limitations.

QOLS is a reliable and valid 16 item generic instrument [34]., and was selected for use as it has been extensively used in chronic conditions and provides a subjective, global evaluation of QOL.

Data on participants' clinical variables, such as frequency of painful crises in past year, haemoglobin levels, serum creatinine and LDH levels, were obtained from their medical records. The study was granted ethical approval by the University of the West Indies/University Hospital of the West Indies, Faculty of Medical Sciences Ethics Committee.

### Statistical approach

All data were initially captured into Epidata^® ^for Windows and then analyzed with Stata™ statistical software for Windows version 8.2 [35].

Domain scores for the WHOQOL were transformed to a 4–20 score according to accepted guidelines [36]. Cronbach's alpha values of .70 and over were deemed acceptable [37]. The floor and ceiling effects were measured for the scales and their domains with floor effect being the percentage of subjects with the lowest possible domain scores and the ceiling effect being the percentage of subjects with the highest possible domain scores.

The psychometric properties were further tested by measuring the "known-groups" construct validity. The presence of painful crises in SCD is a very prevalent and severe complication of the disease [38,39], and those with higher pain rates tend to die earlier than those with lower pain rates [40]. Painful crises were defined as presence of bony pains requiring opioid analgesics for relief, and categorized (less than or equal to 3 episodes per year or greater than 3 episodes for the year). T-test was used to test whether the scores in the three instruments could discriminate among different categories.

Pearson's correlations were used to determine the level of agreement between the three instruments, as well as with markers of disease severity. As a general guideline, correlations from 0.00 to 0.25 indicate little or no relationship, from 0.25 to 0.50 a fair degree of relationship, from 0.50 to 0.75 a moderate to good relationship, and above 0.75 a good to excellent relationship [41].

## Results

### Demographics and clinical characteristics

A total of 491 patients participated (Table 1), consisting of 43% males and 57% females. The mean age was 31.3 years ± 9.6 years with a range from 18–70 years. The commonest genotypes were 68% SS (Homozygous S Disease) disease and 21.5% SC (Heterozygous S-C Disease). Most were 'single' (88%) with only 10% being 'married'. Only 51.5% were employed currently. 54% had a secondary education, 24% had vocational training and 6% had a tertiary education.

**Table 1:** Demographic and clinical characteristics of the study population (n = 491)

**Variable**	
Sex, M: F (%)	210 (42.7): 281 (57.3)
Age, mean years (SD)	31.3 (9.6)
Genotype, %	
SS	68.1
SC	21.5
Others	10.4
Education, (%)	
Primary	72 (14.7)
Secondary	266 (54.2)
Vocational training	119 (24.2)
Tertiary	30 (6.1)
Employment status, Y: N (%)	253 (51.5): 238 (48.5)
Marital Status, (%)	
Single	431 (87.8)
Married	48 (9.8)
Other	12 (2.4)
Haemoglobin g/dl, mean (SD)	9.0 (2.2)
Fetal Haemoglobin %, mean (SD)	4.6 (4.3)
Lactate Dehydrogenase IU/L, mean (SD)	391.73 (193.2)
Serum Creatinine μmol/L, mean (SD)	60.4 (25.4)
Painful Crises, n (%)	
0–3 per year	412 (83.9)
More than 3 per year	79 (16.1)

The mean haemoglobin was 9.0 ± 2.2 gm/dl; and fetal haemoglobin was 4.6 ± 4.3%. The mean serum creatinine and LDH were 60.4 ± 25.4 μmol/L and 391.7 ± 193.2 IU/L respectively. 83.9% had 0–3 painful crises for the past year and 16.1% had greater than 3.

### Psychometric properties of the WHOQOL-Bref, QOLS and SF-36

The baseline means, standard deviations, minimum/maximum and internal consistency reliability coefficients for all three instruments and their domains are summarized in Table 2. All scales had moderate Cronbach's alpha scores, ranging from 0.70 to 0.93, except the WHOQOL- social relationship domain (0.66). The mean scores for the WHOQOL-physical health and WHOQOL-environment were lower than the other domain scores. The SF-36 and QOLS had generally higher reliability coefficients than the WHOQOL-Bref. Most domains had no marked floor or ceiling effects (<1%), exceptions being WHOQOL-social relations (ceiling effect = 3.9%), SF-mental health and SF-role limitations domains (ceiling effects ~19%).

**Table 2:** Descriptive Statistics of all three measures and their domains*

	**Cronbachs Alpha**	**Minimum**	**Maximum**	**Mean**	**Std. Deviation**	**Floor effect (%)**	**Ceiling effect (%)**
WHOQOL-Physical	0.87	7.43	20.00	13.96	2.71	0.2	0.8
WHOQOL-Psychological	0.82	6.67	19.33	14.18	2.12	0.2	0.4
WHOQOL- Social Relations	0.66	5.33	20.00	14.91	2.77	0.2	3.9
WHOQOL-Environment	0.81	7.50	19.00	13.38	2.24	1.0	0.2
Total WHOQOL Score	0.81	34.17	76.34	56.43	7.87	0.2	0.2
SF 36-Physical Health	0.86	10	30	26.46	3.49	0.4	19.1
SF36-Mental Health	0.93	15	45	32.45	6.21	0.2	0.4
SF36-Role Limitations	0.90	12.2	42	34.54	7.13	0.2	19.2
Total SF36 Score	0.70	48.2	117	93.46	13.81	0.2	0.4
QOLS Score	0.88	38	114	78.0	10.8	0.2	0.2

* Higher scores reflect better quality of life on each domain of all measures

Table 3 shows the known-groups validity where the mean scores decreased, meaning lower quality of life on each scale/domain, as frequency of painful crises increased (All p < 0.01 for ANOVA).

**Table 3:** Scale and domain scores for categories of painful crises

	0–3 painful crises/year (N = 412)	>3 painful crises/year (N = 79)	p-value
WHOQOL-Physical	14.3 (14.0, 14.5)	12.3 (11.8,12.8)	<0.001
WHOQOL-Psychological	14.3 (14.1, 14.5)	13.6 (13.1,14.1)	0.009
WHOQOL- Social Relations	15.1 (14.8, 15.3)	14.1 (13.4, 14.7)	0.004
WHOQOL-Environmental	13.5 (13.3, 13.7)	12.5 (11.9, 13.1)	<0.001
Total WHOQOL Score	57.2 (56.4, 57.9)	52.6 (50.9, 54.3)	<0.001
SF 36-Physical Health	26.7 (26.4, 27.0)	25.2 (24.4, 26.0)	<0.001
SF36-Mental Health	33.1 (32.6, 33.7)	28.9 (27.5, 30.3)	<0.001
SF36-Role Limitations	35.6 (35.1, 36.4)	28.2 (26.6, 29.9)	<0.001
Total SF36 Score	95.6 (94.4, 96.8)	82.3 (78.9, 85.7)	<0.001
QOLS Score	78.7 (77.6, 79.7)	74.6 (71.9, 77.1)	0.002

Values are mean (95% C.I.)

### Correlation analyses

Table 4 demonstrates the correlations of the WHOQOL-Bref with SF-36, QOLS and clinical variables. The total SF-36 and WHOQOL-Bref scores had an acceptable positive correlation (0.64). The WHOQOL-Bref domains showed moderate correlations with SF-36-mental health, ranging from 0.51 for WHOQOL-social relationships to 0.59 for WHOQOL-psychological, and with the total SF-36 score (0.47–0.53). They had much stronger correlations with the QOLS score, ranging from 0.43 for WHOQOL-physical to 0.71 for WHOQOL-environmental. The WHOQOL total score correlation with the QOLS score was high at 0.75.

**Table 4:** Correlations between WHOQOL-Bref domains, SF score, QOLS score and clinical variables

	WHOQOL-Physical	WHOQOL-Psychological	WHOQOL- Social Relations	WHOQOL-Environmental	Total WHOQOL Score
SF 36-Physical Health	0.3733**	0.3286**	0.2460**	0.3386**	0.4001**
SF36-Mental Health	0.5200**	0.5895**	0.5100**	0.5862**	0.6844**
SF36-Role Limitations	0.3654**	0.3427**	0.3513**	0.3547**	0.4428**
Total SF36 Score	0.5166**	0.5248**	0.4727**	0.5321**	0.6372**
QOLS Score	0.4251**	0.6552**	0.6492**	0.7130 **	0.7545**
Haemoglobin	0.3444**	0.1908**	0.1607**	0.1752**	0.2761**
Lactate Dehydrogenase	-0.3355**	-0.1202*	-0.2017**	-0.1512**	-0.2550**

* P < 0.05, ** P < 0.01, based on Student's t test

As expected, the clinical variables showed significant correlations with WHOQOL-physical health: -0.34 with LDH and 0.34 with haemoglobin. These variables also had smaller, significant correlations with the total WHOQOL score.

## Discussion

The main purpose of this paper was to assess the utility of this instrument in patients with SCD living in Jamaica. In all of its performance measures, the WHOQOL-Bref has compared favourably with other studies. The Cronbach's alpha for each of its domains were large, except for WHOQOL-Social relations, which is similar to other large, multicentre trials [32], and may be because it consists of only three items. The ceiling effects for WHOQOL-Social relations were also high similar to studies in patients with chronic obstructive airway disease where the ceiling effect was 5.2% [27]. In fact the WHOQOL-Bref showed lower effects than the SF-36, as the latter had high ceiling effects for two of its domains.

The instrument was able to discriminate between groups experiencing different frequencies of painful crises. Pain is a major indicator of health-seeking and hospitalization in these patients [38,39,42-45], and those with frequent painful crises have shown poorer QOL in past studies [9,23]. The WHOQOL-Bref has shown significantly lower scores in those who have more frequent painful crises. This mirrors original work by Skevington [46], which has shown the sensitivity of the WHOQOL instruments to pain states.

The WHOQOL-Bref score had fair convergent validity, and the fact that it did not have stronger correlations with the SF-36 and QOLS suggests that while it does share some overlap with these existing measures, it assesses a unique aspect of quality of life not assessed by the either the SF-36 or the QOL. All domains of the WHOQOL-Bref had greatest correlations with SF-mental health and secondly with the total SF-36 score. This may be due to the fact that SF-physical health is a more objective measure whereas the WHOQOL-physical health is a purely subjective measure. This was mirrored in the study comparing the WHOQOL-Bref with SF-36 in patients with stroke [41], where the SF-physical health showed low correlations with most domains of the WHOQOL.

WHOQOL-physical health has significant correlations with more objective clinical variables, i.e. haemoglobin levels and LDH. Lower haemoglobin and higher LDH levels are known to be associated with more severe SCD experience [44,47-49]. The expected relationships therefore, between WHOQOL-physical health and these clinical parameters have been shown in this study. Similarly, the WHOQOL-psychological health has shown good convergent validity as evidence by its moderate correlation with SF-mental health.

Not unlike past research, the present study has also employed a cross-sectional design to study QOL in SCD, and so is limited in its ability to examine the stability or responsiveness to change in QOL in these patients. Future research could examine how their QOL fluctuates with changes in their health, as well as how the latter affect test-retest reliability of QOL instruments.

In conclusion, the WHOQOL-Bref has shown fairly good utility in this specific disease population. It also compares favourably to other generic instruments to measure QOL such as the SF-36 and QOLS.

## Competing interests

The authors declare that they have no competing interests.

## Authors' contributions

All authors have contributed substantially to study design, data collection, analysis of data and preparation of the manuscript. All authors have also read and approved the final manuscript.
